# Development of a novel scoring system to determine the optimal timing of esophagogastroduodenoscopy following portosystemic shunt occlusion

**DOI:** 10.1371/journal.pone.0341330

**Published:** 2026-01-20

**Authors:** Tatsuro Nishimura, Aika Kirihara, Maho Egusa, Natsuko Nishiyama, Tsuyoshi Fujioka, Daiki Kawamoto, Ryo Sasaki, Norikazu Tanabe, Issei Saeki, Toshihiko Matsumoto, Tsuyoshi Ishikawa, Taro Takami

**Affiliations:** Department of Gastroenterology and Hepatology, Yamaguchi University Graduate School of Medicine, Ube-Yamaguchi, Japan; Al-Azhar University, EGYPT

## Abstract

Balloon-occluded retrograde transvenous obliteration (BRTO) is a safe and effective treatment for gastric varices (GV) and refractory hepatic encephalopathy (HE) associated with portosystemic shunt (PSS). However, esophageal varices (EV) worsening after BRTO is a major, postoperative complication. This study aimed to develop a novel scoring system for predicting EV deterioration following PSS occlusion and to determine the optimal timing for esophagogastroduodenoscopy (EGD) after the operation for patients with portal hypertension. We retrospectively analyzed data from 76 patients with PSS who underwent BRTO for GV or refractory HE [male/female = 39/37; Child-Pugh class A/B/C = 37/32/7; mean Hepatic venous pressure gradient = 10.8 mmHg] at our institution between April 2008 and March 2021. Factors associated with EV deterioration after BRTO were identified statistically. Cumulative rates of EV deterioration were determined using the Kaplan-Meier method. During a median follow-up period of 18.0 months, 50 patients experienced EV deterioration. The median time to EV deterioration was 23.4 months. Cumulative rates of EV deterioration at 12, 24, and 36 months were 35.5%, 51.5%, and 58.1%, respectively. Multivariate analysis using a Cox proportional hazards model identified male sex (*p* = 0.013), a preoperative platelet count ≤ 8.3x10⁴/μL (*p* = 0.032), and presence of EV before BRTO (*p* = 0.029) as significant independent risk factors for postoperative EV deterioration. Based on these three factors, we developed a novel scoring system ranging from 0–3 points. Median time to EV deterioration for scores 0, 1, 2, and 3 was unreached, 19.2, 10.2, and 6.5 months, respectively. Post-BRTO EV deterioration can be predicted using three factors: male sex, a preoperative platelet count ≤ 8.3x10⁴/μL, and the presence of EV before BRTO. This novel scoring system provides a structured approach for determining the optimal timing of EGD after BRTO, allowing risk stratification, improved postoperative patient management, early detection of EV deterioration, and timely endoscopic intervention for high-risk varices.

## Introduction

Balloon-occluded retrograde transvenous obliteration (BRTO) is widely performed in the management of isolated gastric varices (GV) and refractory encephalopathy associated with a portosystemic shunt (PSS) [1–3]. In addition, BRTO has been reported to improve hepatic functional reserve and long-term patient outcomes by fully occluding the PSS [4–6]. A previous study demonstrated that pre-BRTO liver stiffness levels, assessed via transient elastography, serve as an independent prognostic predictor of BRTO. Lower preprocedural liver stiffness levels may also correlate with a reduced incidence of postprocedural adverse events related to secondary elevations in portal venous pressure following PSS occlusion [7].

In clinical practice, particular attention must be given to esophageal varices (EV) deterioration after BRTO, as it is one of the most concerning postoperative complications. The cumulative rates of post-BRTO EV deterioration range from 66–68% [8–10]. Although some studies have explored the predictive factors for EV deterioration, no scoring system has been developed to assess the risk of deterioration. Furthermore, esophagogastroduodenoscopy (EGD) for EV is recommended every 1–2 years for patients with liver cirrhosis (LC) [11]; however, to the best of our knowledge, the optimal post-BRTO EGD follow-up interval remains unclear.

Thus, the primary aim of this study was to develop a novel scoring system for predicting EV deterioration after BRTO. The secondary aim was to determine the optimal timing for EGD following treatment.

## Methods

### Patients

**Fig 1:**
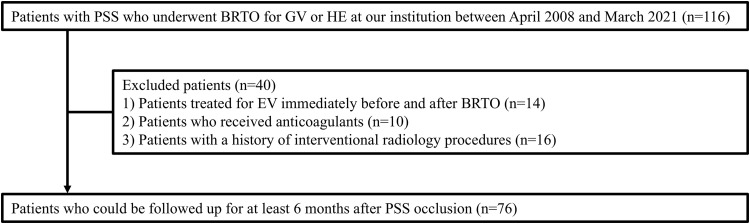
Flowchart of patient selection for this study. GV, gastric varices; HE, hepatic encephalopathy; BRTO, balloon-occluded retrograde transvenous obliteration; EV, esophageal varices; PSS, portosystemic shunt.

This single-center study retrospectively analyzed existing data of 116 patients with LC who had GV or hepatic encephalopathy (HE) with PSS, such as a gastrorenal or splenorenal shunt, who underwent BRTO at our institution between April 1, 2008, and March 31, 2021. The database was accessed after May 2, 2022, when the study was approved by the ethics committee, and all data were collected following anonymization. Contraindications of BRTO in our institute were obstruction of the portal vein trunk and/or splenic vein and refractory ascites. Biochemical, clinical, and ultrasonographic findings confirmed the diagnosis of LC. Fourteen patients who were treated for EV immediately before and after BRTO were excluded, because endoscopic treatment can alter variceal morphology and obscure the assessment of BRTO-related hemodynamic effects on subsequent EV deterioration. Ten patients who received anticoagulants, including warfarin, were excluded from the analysis because their prothrombin time (PT) percentage activities and PT-international normalized ratio were unsuitable for Child-Pugh (CP) and Model for End-Stage Liver Disease-Sodium (MELD-Na) score calculations. Sixteen patients with a history of interventional radiology procedures, such as partial splenic embolization and transjugular intrahepatic portosystemic shunt, were also excluded because these procedures could affect portal-splenic venous hemodynamics. Finally, data from 76 patients were analyzed and followed up for at least 6 months after PSS occlusion (Fig 1). The study was conducted in accordance with the guidelines of the Declaration of Helsinki, and the protocol was approved by the Institutional Review Board of Yamaguchi University Hospital (approval number: 2022−013; date of approval: May 2, 2022). Informed consent for the disclosure of this study was obtained from all patients, with an opt-out option. Further informed consent was waived by the Institutional Ethics Committee.

### BRTO procedure

The BRTO procedure was performed using the method described by Kanagawa et al.^1^ and other previously described procedural protocols [7,12–14]. Briefly, a balloon catheter was inserted into the gastrorenal or splenorenal shunt via the left renal vein, and blood flow was occluded by inflating the balloon. Following balloon-occluded retrograde transvenous venography, 5% ethanolamine oleate with iopamidol was slowly injected until it remained in the splenorenal shunt in the HE cases, or until the GV and the feeding veins were visualized in the GV cases. The inflated balloon catheter was left in place overnight. If thrombosis was confirmed the following day under fluoroscopy, the balloon catheter was deflated and removed.

### Biochemical and diagnostic imaging assessments

Hepatic function markers, including total bilirubin and albumin levels, and PT percentage activities, were evaluated within 3 days before BRTO. In addition, platelet count, electrolyte levels (sodium), renal function (creatinine levels), and hepatic fibrosis markers and indices, such as the 7S domain of type IV collagen, aspartate aminotransferase-to-platelet ratio index, and fibrosis-4 index, were evaluated. CP, MELD-Na, and albumin-bilirubin scores were calculated before BRTO. The liver and spleen volumes were measured prior to the procedure based on previous reports [12–14]. Liver stiffness was measured by TE using the FibroScan system (Echosens SA, Paris, France) before BRTO [15].

### Measurements of hepatic venous pressure gradient

Before BRTO, wedged hepatic venous pressure (WHVP) was measured and the hepatic venous pressure gradient (HVPG) was calculated, as described previously [12–14,16]. Briefly, the right hepatic venous branch was catheterized, and the free hepatic venous pressure and WHVP were measured using diluted contrast medium before and after vein occlusion; this was achieved by inflating a balloon catheter (Terumo Clinical Supply Co., Ltd., Gifu, Japan). The HVPG was defined as the pressure difference between the portal and hepatic veins, and it was calculated by subtracting the free hepatic venous pressure from the WHVP.

### Endoscopic evaluation of EV

Endoscopic findings of EV were evaluated by experienced endoscopists based on the criteria proposed by the Japan Society for Portal Hypertension (PH) [17]. The GV location was classified into three groups: Lg-c, adjacent to the cardiac orifice; Lg-cf, extending from the cardiac orifice to the fornix; or Lg-f, localized to the fornix. GV and EV were classified into three groups: F1, straight or relatively small-caliber varices; F2, moderately enlarged or beady varices; and F3, markedly enlarged, nodular, or tumor-shaped varices. Red color (RC) signs, including red wale markings, cherry red spots, and hematocystic spots, are recognized as reliable predictors of variceal bleeding. These signs refer to reddish changes observed just beneath the submucosa [18,19]. Deterioration of the EV was defined as rupture of the EV, appearance of RC signs, or at least one-step deterioration of the F category compared with the EGD findings before BRTO. After treatment, EGD was performed at 1–2 months and then every 6 months if high risk of EV bleed was absent. In Japan, F2 and F3 EV with RC signs were associated with a high risk of bleeding. Patients were followed from the date of BRTO until the first EGD documenting EV deterioration or censoring. Patients with no deterioration of EV were censored on the date of their last EGD, and only those who could be followed for at least 6 months after BRTO were included in the analysis. Death and hepatic failure were considered potential competing events against EV deterioration; however, no patient experienced these events before the last EGD during the observation period; hence, the impact of competing risk bias was considered minimal.

### Statistical analyses

Data are expressed as means with standard deviations or medians with ranges. The JMP software (version 16; SAS Institute Inc., Cary, NC, USA) was used for statistical analyses. Long-term prognosis after BRTO was evaluated by overall survival (OS) and liver-related events (LREs). OS, estimated using the Kaplan–Meier method, was defined as the period from BRTO to death from any cause or last follow-up. LREs included ascites, overt hepatic encephalopathy, variceal bleeding, acute-on-chronic liver failure, and hepatocellular carcinoma. Their cumulative incidence was calculated from BRTO to the first event and illustrated as a cumulative incidence curve. The cumulative rates of EV deterioration post-BRTO were estimated using the Kaplan–Meier method. A receiver operating characteristic curve was drawn using JMP. The area under the receiver operating characteristic curve was used to evaluate the ability of a factor to predict EV deterioration after BRTO, and the optimal cutoff value for each predictor was determined, which was used to divide patients into two groups. Univariate and multivariate Cox proportional hazards analyses were used to assess the association between the baseline variables and EV deterioration after BRTO. Hazard ratio (HR), 95% confidence interval (CI), and *p*-values were calculated. The log-rank test was used to determine the differences in cumulative EV deterioration rates for each factor. Statistical significance was set at *p* < 0.05.

## Results

### Baseline clinical characteristics of patients

The baseline clinical characteristics of the patients are summarized in Table 1. The patients included 39 males and 37 females with a mean age of 67.6 ± 8.8 years. The causes of cirrhosis included hepatitis B (*n* = 3), hepatitis C (*n* = 27), Alcohol-associated liver disease (ALD) (*n* = 23), and metabolic dysfunction-associated steatohepatitis (*n* = 13). The indications for BRTO were GV in 60 patients and HE in 16. Ten patients had a history of EV treatment. The most common EV form before BRTO was F1 in 31 patients, and no RC sign was observed. Eight patients had coexisting hepatocellular carcinoma at the time of BRTO. The mean preprocedural CP score was 6.9 ± 1.8, and most patients (*n* = 37) were classified as CP class A. The mean preprocedural MELD-Na and albumin-bilirubin scores were 11.0 ± 3.1 and −1.9 ± 0.5, respectively.

**Table 1:** Baseline clinical characteristics of patients.

Characteristics	n = 76
Age (years)	67.6 ± 8.8
Sex: male/female	39/37
Etiology of cirrhosis: HBV/HCV/ALD/MetALD/MASH/others	3/27/23/0/13/10
Therapeutic indication: GV/HE	60/16
History of EV treatment: presence/absence	10/66
GV form: F1/F2/F3	0/42/18
GV location: Lg-c/Lg-f/Lg-cf	4/17/39
EV form before BRTO: F1/F2/F3	31/6/0
HCC: presence/absence	8/68
Liver volume (cm³)	1006.9 ± 326.4
Spleen volume (cm³)	321.2 ± 208.0
Liver stiffness (kPa)	23.5 ± 15.9
HVPG (mmHg)	10.8 ± 3.5
Child-Pugh score	6.9 ± 1.8
Child-Pugh class: A/B/C	37/32/7
MELD-Na score	11.0 ± 3.1
ALBI score	−1.9 ± 0.5
Albumin (g/dL)	3.4 ± 0.5
Total bilirubin (mg/dL)	1.3 ± 0.6
Creatinine (mg/dL)	0.8 ± 0.8
Sodium (mmol/L)	138.8 ± 2.3
Platelet count (x10⁴/μL)	11.0 ± 4.9
PT (%)	71.9 ± 18.5
PT-INR	1.2 ± 0.2
IV COL-7S (ng/mL)	8.9 ± 2.9
APRI	1.4 ± 1.1
FIB-4 index	5.7 ± 3.0

HBV, hepatitis B virus; HCV, hepatitis C virus; ALD, Alcohol-associated liver disease; MetALD, Metabolic Alcoholic Liver Disease; MASH, metabolic dysfunction-associated steatohepatitis; GV, gastric varices; HE, hepatic encephalopathy; EV, esophageal varices; BRTO, balloon-occluded retrograde transvenous obliteration; HCC, hepatocellular carcinoma; HVPG, hepatic venous pressure gradient; MELD-Na, model for end-stage liver disease; ALBI, albumin-bilirubin; PT, prothrombin time; INR, international normalized ratio; IV COL-7S, 7S domain of type IV collagen; APRI, aspartate aminotransferase-to-platelet ratio index; FIB-4, fibrosis-4 index.

Dates are presented as means and standard deviations, unless otherwise specified.

#### Long-term prognosis after BRTO.

**Fig 2:**
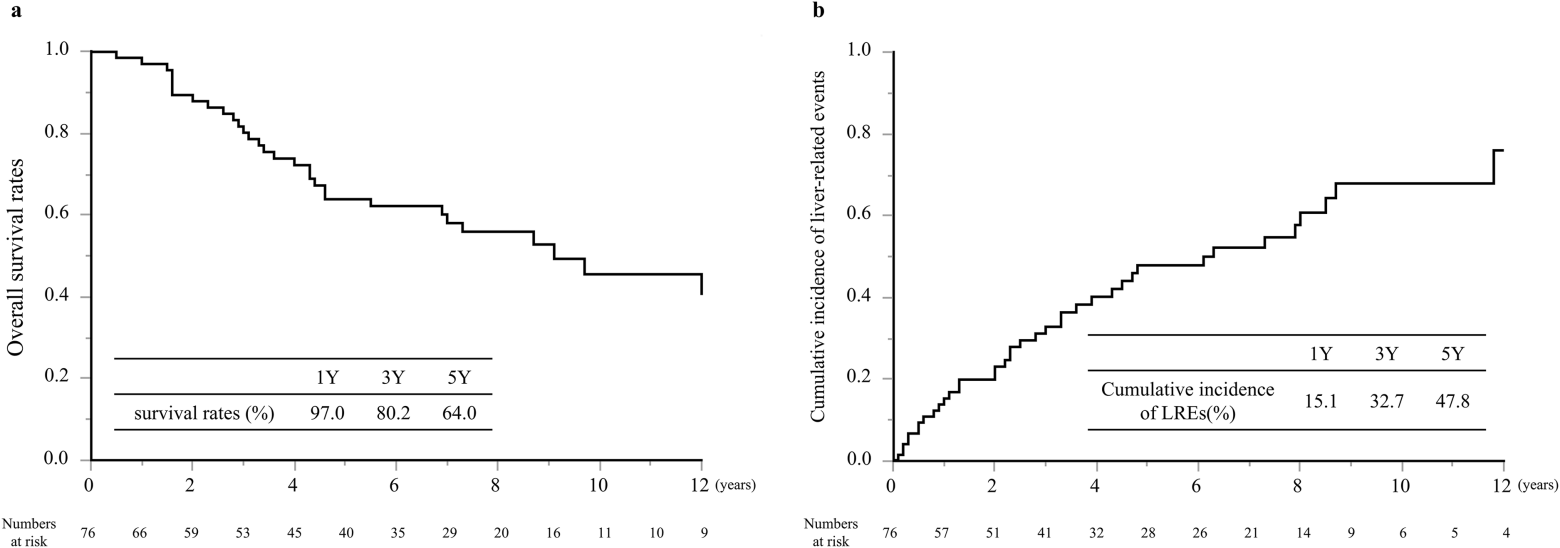
(a) Overall survival after BRTO, (b) Cumulative incidence of liver-related events after BRTO. BRTO, balloon-occluded retrograde transvenous obliteration; LREs, liver-related events.

Overall survival after BRTO was generally favorable in this cohort. The Kaplan–Meier analysis showed 1-, 3-, and 5-year OS rates of 97.0%, 80.2%, and 64.0%, respectively (Fig 2a). During follow-up, 31 patients (40.8%) died, of whom 22 (71.0%) experienced liver-related death, mainly due to liver failure (13 patients, 41.9%) and hepatocellular carcinoma (9 patients, 29.0%) (Table 2). We also assessed the long-term occurrence of liver-related events. The cumulative incidence of LREs at 1, 3, and 5 years after BRTO was 15.1%, 32.7%, and 47.8%, respectively (Fig 2b). These results suggest that, although overall survival after BRTO is favorable, a substantial proportion of patients develop liver-related complications over time, reflecting the progressive course of the underlying liver disease.

**Table 2:** Causes of death after BRTO.

Cause of death	Total (n = 31)
Liver-related death	22 (71.0%)
Liver failure	13 (41.9%)
HCC	9 (29.1%)
Extrahepatic cancers	4 (12.9%)
Renal failure	1 (3.2%)
Respiratory failure	1 (3.2%)
Unknown	3 (9.7%)

BRTO, balloon-occluded retrograde transvenous obliteration; HCC, hepatocellular carcinoma.

### Cumulative rates of EV deterioration after BRTO

**Fig 3:**
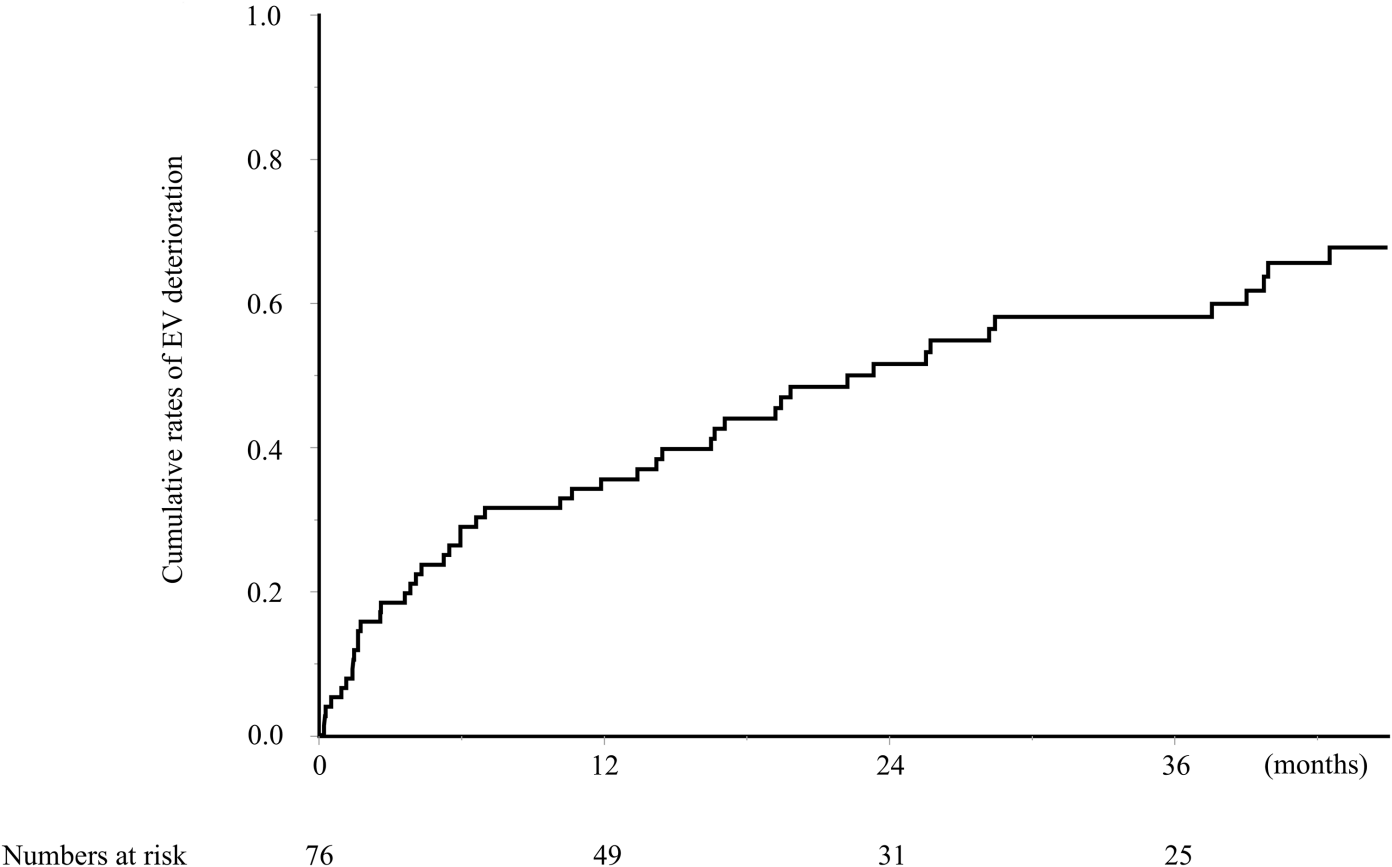
Cumulative rates of EV deterioration after BRTO. BRTO, balloon-occluded retrograde transvenous obliteration; EV, esophageal varices.

BRTO was successfully performed in 76 patients. The median follow‑up duration was 18.0 months (interquartile range: 5.9–39.6). Thus, although follow‑up times varied, observation periods for most patients were clinically meaningful for assessing EV deterioration after BRTO. Deterioration of EV was observed in 50 patients, of whom 42 showed deterioration of the F category and 5 showed appearance of RC signs. Rupture of the EV after BRTO was observed in three patients, with a time to rupture of 7.9, 10.7, and 27.6 months. The median time to EV deterioration was 23.4 months (range, 1–59 months). The cumulative rates of EV deterioration at 12, 24, and 36 months were 35.5%, 51.5%, and 58.1%, respectively (Fig 3).

### Predictive factors of EV deterioration after BRTO

**Fig 4:**
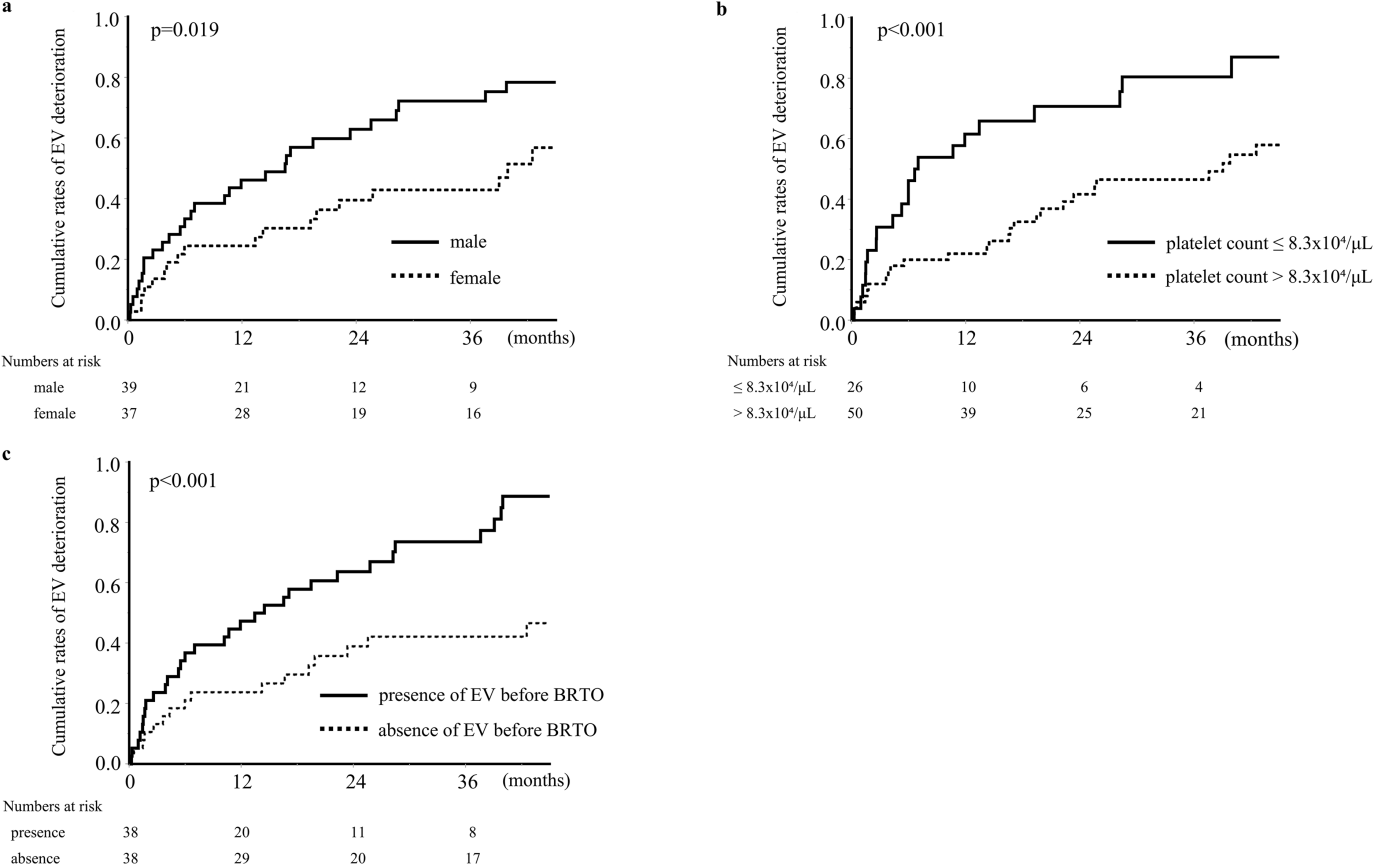
Comparison of the cumulative rates of EV deterioration after BRTO according to (a) Sex, (b) Preoperative Platelet Count, and (c) EV Before BRTO. BRTO, balloon-occluded retrograde transvenous obliteration; EV, esophageal varices.

The univariate analysis using a Cox proportional hazards model revealed a significant association between EV deterioration after BRTO and male sex, a preoperative platelet count ≤ 8.3x10⁴/μL, a preoperative MELD-Na score of >11 and the presence of EV before BRTO (Table 3). The multivariate analysis revealed that male sex (HR: 2.27, 95% CI: 1.17–4.39, *p=*0.013), a preoperative platelet count ≤ 8.3x10⁴/μL (HR: 2.11, 95% CI: 1.06–4.19, *p* = 0.032), and the presence of EV before BRTO (HR: 2.36, 95% CI: 1.06–5.27, *p* = 0.029) were significant independent risk factors of EV deterioration. The rates of EV deterioration were significantly higher in patients with male sex, a preoperative platelet count ≤ 8.3x10⁴/μL, and the presence of EV before BRTO, relative to other populations (Fig 4). To further justify this platelet threshold, ROC analysis for EV deterioration within 1 year after BRTO showed that a cutoff of 8.3 × 10⁴/μL yielded an AUC of 0.69, with a sensitivity of 79.6%, a specificity of 59.3%, and a Youden index of 0.39.

**Table 3:** Predictive factors of EV deterioration after BRTO.

Variables	Univariate analysis			Multivariate analysis		
	HR	95% CI	*p*-value	HR	95% CI	*p*-value
Age: > 68 vs. ≤ 68 (years)	1.28	0.73-2.24	0.395			
Sex: male vs. female	1.96	1.11-3.48	0.019	2.27	1.17–4.39	0.013
Etiology of cirrhosis: viral vs. nonviral	1.37	0.76-2.46	0.683			
Therapeutic indication: GV vs. HE	1.08	0.57-2.08	0.806			
History of EV treatment: presence vs. absence	1.84	0.90-3.82	0.118			
EV before BRTO: presence vs. absence	2.91	1.61-5.30	<0.001	2.36	1.06–5.27	0.029
HCC: presence vs. absence	1.68	0.89-3.17	0.131			
Liver volume: ≤ 947 vs. > 947 (cm³)	1.33	0.76-2.32	0.323			
Spleen volume: > 256 vs. ≤ 256 (cm³)	1.29	0.73-2.28	0.382			
Liver stiffness: > 16.9 vs ≤ 16.9 (kPa)	1.81	0.89-3.65	0.084			
HVPG: > 10 vs ≤ 10 (mmHg)	1.51	0.68-3.35	0.304			
Child-Pugh score: > 7 vs. ≤ 7	1.44	0.81-2.57	0.155			
MELD-Na score: > 11 vs. ≤ 11	1.96	1.06-3.61	0.029	1.12	0.56–2.22	0.747
ALBI score: ≤ −2.0 vs. > -2.0	1.41	0.79-2.54	0.239			
Albumin: ≤ 3.5 vs. > 3.5 (g/dL)	1.63	0.89-2.99	0.109			
Total bilirubin: > 2.0 vs. ≤ 2.0 (mg/dL)	1.61	0.76-3.44	0.235			
Creatinine: > 0.82 vs. ≤ 0.82 (mg/dL)	1.31	0.70-2.43	0.303			
Sodium: ≤ 137 vs. > 137 (mmol/L)	1.51	0.78-2.91	0.213			
Platelet count: ≤ 8.3 vs. > 8.3 (x10⁴/μL)	2.48	1.40-4.38	<0.001	2.11	1.06–4.19	0.032
PT: ≤ 70 vs. > 70 (%)	1.03	0.58-1.84	0.901			
PT-INR: > 1.5 vs. ≤ 1.5	1.33	0.53-3.38	0.542			
IV COL-7S: > 8.6 vs. ≤ 8.6 (ng/mL)	1.41	0.73-2.72	0.204			
APRI: > 0.85 vs. ≤ 0.85	1.11	0.62-1.98	0.675			
FIB-4 index: > 4.0 vs. ≤ 4.0	1.69	0.91-3.15	0.093			

EV, esophageal varices; BRTO, Balloon-occluded retrograde transvenous obliteration; HR, hazard ratio; CI, confidence interval; GV, gastric varices; HE, hepatic encephalopathy; HCC, hepatocellular carcinoma; HVPG, hepatic venous pressure gradient; MELD-Na, Model for End-Stage Liver Disease-sodium; ALBI, albumin-bilirubin; PT, prothrombin time; INR, international normalized ratio; IV COL-7S, 7S domain of type IV collagen; APRI, aspartate aminotransferase-to-platelet ratio index; FIB-4, fibrosis-4 index.

### Prediction score for EV deterioration after BRTO

**Fig 5:**
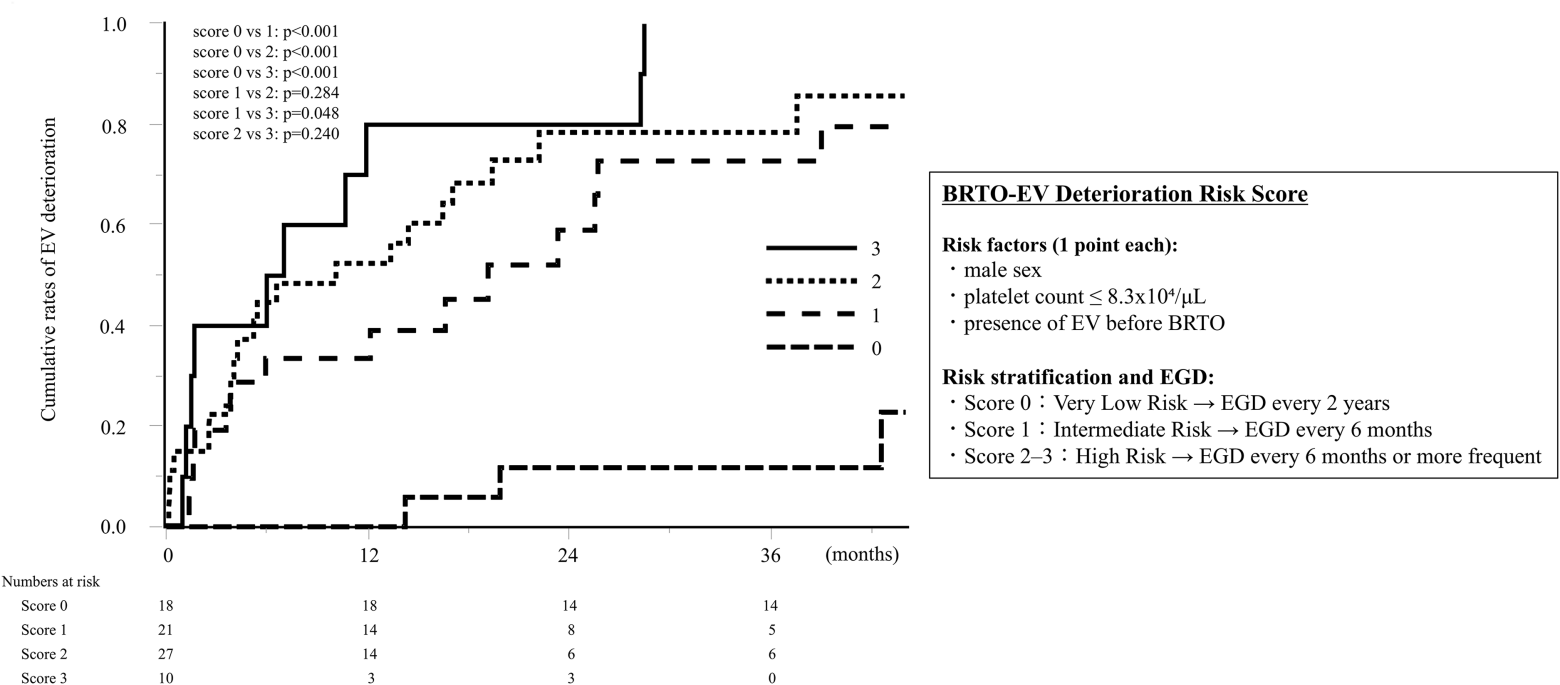
Cumulative rates of EV deterioration based on score. EV, esophageal varices.

A novel scoring system was developed for predicting EV deterioration after BRTO based on the significant variables obtained from the multivariate analysis. This scoring system consisted of three factors, namely sex, preoperative platelet count, and the presence of EV before BRTO, which was calculated as follows: sex (male = 1, female = 0), platelet count (≤8.3x10⁴/μL = 1, > 8.3x10⁴/μL = 0), and EV before BRTO (presence = 1, absence = 0). Since the HR of each factor was approximately equal, we assigned one point to each factor (range, 0–3 points). The patients were stratified into four groups according to this scoring system and showed significantly different cumulative rates for EV deterioration (*p* < 0.001). The median time to EV deterioration at 0, 1, 2, and 3 points was unreached, 19.2 (range, 2–50) months, 10.2 (range, 1–59) months, and 6.5 (range, 1–29) months, respectively (Fig 5).

### Comparison of postoperative clinical course between two patients with different prediction scores for EV deterioration after BRTO

**Fig 6:**
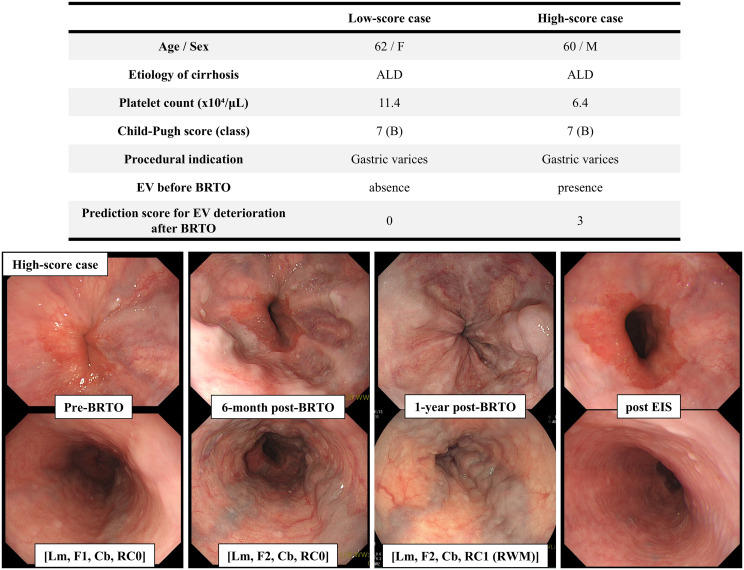
Comparison of postoperative clinical course between two patients with different prediction scores for EV deterioration after BRTO. The patient with a score of 3 shown in underwent esophagogastroduodenoscopy every 6 months, which allowed early detection of EV deterioration and prophylactic treatment. BRTO, balloon-occluded retrograde transvenous obliteration; EV, esophageal varices; ALD, Alcohol-associated liver disease; EIS, endoscopic injection sclerosis; RC, Red color.

Fig 6 shows a comparison of the postoperative clinical course between two patients who underwent BRTO for GV at our institution with low and high prediction scores for EV deterioration (note: these cases were not included in the present retrospective study). In both cases, the etiology of cirrhosis was ALD, and the preoperative CP score was 7; however, the predictive scores differed, wherein one patient had a score of 0 and the other had a score of 3. In the low-scoring case, the EV did not deteriorate for 2 years; however, in the high-scoring case, the EV deteriorated from F1 to F2 at 6 months post-BRTO. After strict follow-up, the patient showed signs of RC at 1 year post-BRTO. Endoscopic injection sclerotherapy was performed, and no recurrences of EV were observed 1 year post-treatment. These illustrative cases were used solely to demonstrate a possible real-world application of our scoring system and follow-up strategy and were not included in any statistical analyses or in the derivation of the proposed recommendations.

## Discussion

This study demonstrated that three factors, namely sex, preoperative platelet count, and the presence of EV before BRTO, can predict EV deterioration post-BRTO in patients with PSS due to PH. We developed a novel scoring system for predicting EV deterioration post-BRTO, consisting of three factors, and established risk stratification and postoperative follow-up strategies.

Deterioration of EV has been reported as a postprocedural complication that is reflective of elevated portal venous pressure following BRTO. The mechanism of BRTO-related EV deterioration involves changes in portal hemodynamics, namely an increase in portal venous flow, followed by an elevation of portal venous pressure associated with shunt occlusion. Patients undergoing BRTO often have large, native portosystemic shunts, and occlusion of these shunts redirects a substantial volume of blood back into the portal system, leading to acute changes in portal hemodynamics. This situation differs from the usual progression of varices in patients with portal hypertension who do not undergo BRTO, for whom variceal worsening is driven by long-term elevation of portal pressure and gradual remodeling of collateral circulation. The rate of EV deterioration after BRTO has been reported to be 26–35% at 12 months and 66–68% cumulatively [3,8–10]. These findings revealed that the cumulative rates of EV deterioration at 12, 24, and 36 months were 35.5%, 51.5%, and 58.1%, respectively, which are comparable to previous reports [3,8–10]. This is significantly higher than the annual EV deterioration rate of 9% during the natural course of disease progression in patients with LC [20]. Since variceal hemorrhage may lead to high rebleeding and mortality rates, it is important to evaluate the risk of EV deterioration after BRTO and detect EV deterioration as early as possible.

The multivariate analysis showed that EV deterioration post-BRTO was significantly associated with male sex, a preoperative platelet count ≤ 8.3x10⁴/μL, and the presence of EV before BRTO. We developed a novel scoring system for predicting EV deterioration post-BRTO based on these three factors, which are routinely obtained in clinical practice.

Merli et al. reported a significant association between male sex and bleeding from EV [20]. Shinkai et al. also identified male sex as a significant risk factor for EV deterioration after BRTO [21]. These findings consistently suggest that sex-related factors may play an important role in the clinical course of EV. In a subanalysis of our cohort, we also evaluated the potential influence of alcohol consumption; however, no significant differences were observed. Although sex hormones and differences in hemodynamic responses may contribute to this relationship, the precise mechanisms underlying the link between male sex and EV deterioration remain unclear and warrant further investigation. Several studies have demonstrated that platelet count is a useful surrogate marker for PH, as it not only predicts the presence of EV but is also inversely correlated with the severity of EV [22–25]. Therefore, it is speculated that thrombocytopenia may affect the EV deterioration. Several previous studies have also reported platelet thresholds in a similar range, showing that a platelet count below 8.8 × 10⁴ is the only independent factor associated with the presence of large esophageal varices, and that a cutoff of 8.2 × 10⁴/μL is a useful noninvasive predictor of esophageal varices in patients with liver cirrhosis. These findings support the validity of our platelet cutoff of 8.3 × 10⁴/μL in the present study [26–27]. Ninoi et al. showed that patients with EV before BRTO experienced postoperative deterioration of EV approximately five times more often than patients without EV [8]. The presence of EV on EGD before BRTO has been reported to be a risk factor for the deterioration of EV after treatment [20–25,28], consistent with our results.

Here, we present a strategy that utilizes the novel scoring system to determine the optimal timing of EGD monitoring. The median time to EV deterioration at 0, 1, 2, and 3 points was unreached, 19.2, 10.2, and 6.5 months, respectively. These findings suggest that patients with a score of 0 can be considered very low risk, and post‑BRTO EGD every 1–2 years appears sufficient. This schedule is compatible with current concepts of risk‑adapted surveillance in compensated cirrhosis without high‑risk varices and with the absence of clinically relevant early deterioration in this very‑low‑risk population [11]. In contrast, patients with a score of 1 represent an intermediate‑risk group, among whom the median time to EV deterioration was 19.2 months. However, approximately one‑third of EV deteriorations occurred within the first 12 months of follow‑up, suggesting that EGD performed every 6 months would detect most clinically meaningful progression before the onset of variceal bleeding. Patients with scores of 2 or 3 constitute a high‑risk group with the earliest and highest rates of EV deterioration; all post‑BRTO variceal ruptures in our cohort occurred in this category. Therefore, EGD every 6 months, or even more frequently, depending on the overall clinical status and bleeding risk, seems warranted to maintain high sensitivity for capturing high‑risk changes. In our cohort, among patients with a score ≥1, 42.2% of EV deteriorations occurred within 6 months and 54.0% within 12 months after BRTO, indicating that 6‑monthly EGD would capture a substantial proportion of clinically important worsening before potential rupture. The time to rupture for the three patients who experienced EV rupture after BRTO was 7.9, 10.7, and 27.6 months. Their scores were 2, 3, and 2, respectively; notably, all had pre-existing EV before BRTO. These findings suggest that patients with a score ≥2, particularly those with EV before BRTO, may require even stricter postoperative monitoring, whereas a 6-month interval can be regarded as a pragmatic minimum to reduce the likelihood of missing high-risk changes that could precede bleeding. The patient with a score of 3 (Fig 6) underwent EGD every 6 months, which allowed early detection of EV deterioration and prophylactic treatment. Although a formal health economic evaluation was not conducted in this study, this risk‑based follow-up strategy may still improve the feasibility and potential cost-effectiveness of post‑BRTO surveillance by extending surveillance intervals for patients with a score of 0 to reduce unnecessary procedures and related costs, while prioritizing more intensive EGD for higher‑risk patients, especially those with a score ≥2, to help prevent severe variceal bleeding, hospitalizations, and downstream healthcare expenditures.

To the best of our knowledge, this is the first study to determine the appropriate timing of EGD to detect EV deterioration post-BRTO using a novel scoring system for predicting EV deterioration.

Despite the promising results, this study has limitations. First, this was a single-center retrospective study with a relatively small number of patients, and the risk score was developed and evaluated within this single cohort without external validation. Second, HVPG, the gold standard for the diagnosis of PH, and LS, a surrogate marker of HVPG, were not measured in all patients and were not significant factors in this analysis. Third, a formal health economic or cost-effectiveness evaluation of the proposed endoscopic surveillance strategy was not performed, thus, the feasibility of implementing relatively frequent EGD in different healthcare systems remains uncertain. Moreover, our cohort consisted mainly of Japanese patients with cirrhosis due to viral hepatitis or alcohol use, with a mean HVPG of 10.8 mmHg and predominantly Child–Pugh class A/B, reflecting a relatively compensated portal hypertension population undergoing BRTO. Therefore, caution is warranted when extrapolating these findings to Western cohorts with a higher prevalence of MASLD-related cirrhosis or to patients with more advanced liver dysfunction. In addition, patients who were treated for EV immediately before and after BRTO were excluded, which may have led to a slight underestimation of the absolute incidence of EV deterioration. Taken together, these limitations highlight the need for extensive, long-term prospective studies and external validation in independent, preferably multicenter cohorts with diverse etiologies and disease severities to verify our results and further refine the proposed scoring system and surveillance strategy.

## Conclusion

EV deterioration after BRTO was predicted by three factors: male sex, a preoperative platelet count ≤ 8.3x10⁴/µL, and the presence of EV before BRTO. We developed a novel scoring system to predict the deterioration of EV post-BRTO, enabling risk stratification and guiding postoperative management of patients. This system facilitates early detection of EV deterioration and timely endoscopic intervention for high-risk varices.
